# Supplementary material for: Polyketide synthase gene clusters in the ericoid mycorrhizal fungus Oidiodendron maius: genome-wide prediction, expression profiling, and functional characterization of knockout mutants
Source: Front Plant Sci. 2026 Sep 16;17:1924430. doi: 10.3389/fpls.2026.1924430 (PMC13623903; doi:10.3389/fpls.2026.1924430)
Supplement: Supplementary file 1 [file SupplementaryFile1.zip › Supplementary Materials/Supplementary Folder 1.zip!/Oidma1_AssemblyScaffolds_Repeatmasked/knownclusterblast/cluster27/jgi.p_Oidma1_167368_mibig_hits.html]

| MIBiG Protein | Description | MIBiG Cluster | MiBiG Product | % ID | % Coverage | BLAST Score | E-value |
| --- | --- | --- | --- | --- | --- | --- | --- |
| ABC87521.1 | unknown | BGC0001011 | Meridamycin\_biosynthetic\_gene\_cluster | 30 | 100.277777778 | 124 | 2.4e-28 |
| XP\_011392733.1 | Ustilagic\_Acid\_hydroxylase | BGC0001281 | Ustilagic\_acid\_biosynthetic\_gene\_cluster | 29 | 76.6666666667 | 100 | 4.9e-21 |
| ABK64183.1 | reductase | BGC0001247\_c6 | Cercosporin\_biosynthetic\_gene\_cluster | 27 | 103.055555556 | 94 | 3.5e-19 |
| AAM12919.1 | MupF | BGC0000182 | Mupirocin\_biosynthetic\_gene\_cluster | 25 | 99.1666666667 | 73 | 4.9e-13 |
